# Numerical Investigation on the Thermal Performance of Nanofluid-Based Cooling System for Synchronous Generators

**DOI:** 10.3390/e21040420

**Published:** 2019-04-19

**Authors:** Kai Xiong, Yunhua Li, Yun-Ze Li, Ji-Xiang Wang, Yufeng Mao

**Affiliations:** 1 School of Automation Science and Electrical Engineering, Beihang University, Beijing 100191, China; 2 School of Aeronautic Science and Engineering, Beihang University, Beijing 100191, China; 3 Advanced Research Center of Thermal and New Energy Technologies, Xingtai Polytechnic College, Xingtai 054035, China; 4 Institute of Engineering Thermophysics, North China University of Water Conservancy and Electric Power, Zhengzhou 450045, China

**Keywords:** nanofluid-based cooling method, synchronous generator, thermal performance analysis, efficiency analysis

## Abstract

This paper presents a nanofluid-based cooling method for a brushless synchronous generator (BLSG) by using Al_2_O_3_ lubricating oil. In order to demonstrate the superiority of the nanofluid-based cooling method, analysis of the thermal performance and efficiency of the nanofluid-based cooling system (NBCS) for the BLSG is conducted along with the modeling and simulation cases arranged for NBCS. Compared with the results obtained under the base fluid cooling condition, results show that the nanofluid-based cooling method can reduce the steady-state temperature and power losses in BLSG and decrease the temperature settling time and changing ratio, which demonstrate that both steady-state and transient thermal performance of NBCS are improved as nanoparticle volume fraction (NVF) in nanofluid increases. Besides, although the input power of cycling pumps in NBCS has ~30% increase when the NVF is 10%, the efficiency of the NBCS has a slight increase because the 4.1% reduction in power loss of BLSG is bigger than the total incensement of input power of the cycling pumps. The results illustrate the superiority of the nanofluid-based cooling method, and it indicates that the proposed method has a broad application prospect in the field of thermal control of onboard synchronous generators with high power density.

## 1. Introduction

As modern aircraft technology is advancing towards more electrical and all electrical modes [1,2], the demand for electric power in aircraft is steadily increasing. Thus, the required output power of the onboard generator, which is the power source in aircraft, has increased accordingly [3,4]. In order to obtain a relatively small compensatory loss of the generator in aircraft, generators with small volume but high power density, such as brushless synchronous generator (BLSG), are widely deployed in modern aircraft [5]. However, a high power density inevitably leads to a high power loss that is converted to waste heat, resulting in huge heat dissipation requirement. What is worse, the small volume hinders massive heat dissipation for the limited heat removal area. Thus, the issue of thermal protection will become an increasingly critical concern for its operation [6], where an effective heat dissipation method and thermal analysis based on that heat dissipation method are critically needed for BLSG operation.

In order to remove waste heat from the electrical rotating machine effectively and maintain the operating temperature in an acceptable range, various heat removal technologies are applied to the heat dissipation of the electrical rotating machine. In general, three common cooling methods for thermal management of the electrical rotating machine were studied: (1) air cooling [7,8,9], (2) PCM-based cooling [10,11,12], and (3) liquid cooling [13,14,15,16,17]. Nakahama et al. [7] proposed a unidirectional cooling airflow for the thermal protection of an open-type motor that is installed in the electric vehicle. Gorbler et al. [8] conducted a thermal model for an air-cooled high speed PMSM magnet. However, the air cooling method fails to cool the high heat density devices owing to the thermal capacity is extremely limited. PCM-based cooling is also widely used for motor cooling. Wang et al. [9] analyzed the transient cooling effect for a PCM-based cooling permanent magnet synchronous motor; Wang et al. [10] conducted an experimental investigation on the thermal effect of a PCM-based cooling permanent magnet synchronous motor. In spite of its excellent cooling performance, PCM-based cooling is only properly suitable for intermittent working machines. The liquid cooling technologies using oil or water as the working medium are competent for the heat dissipation task where large and continuous heat generation is involved. Specifically, oil spray cooling has been recommended by many researchers for the thermal protection of the high power density electrical machine because of the inherent advantages of spray cooling technology, such as large thermal capacity, high specific surface area of the droplet, and low coolant flow rate. Lim et al. [13] developed an optimized channel for the oil spray cooling model and evaluated its thermal performance suitable for continuous rating condition. Sikora et al. [17] proposed an unconventional water cooling method for medium-power synchronous generators. Though the liquid cooling technology is able to cope with the large heat dissipation mission at present, it may become more and more difficult to satisfy the increasing cooling requirement, especially as the power demand is growing rapidly as the modern aircraft develops further, which will inevitably lead to an increase in power loss and waste heat. Thus, a method to enhance the heat dissipation ability of the liquid cooling is becoming more and more sought-after.

Nanofluids have superior heat dissipation application compared to the base fluid. Therefore, it has been widely studied and gradually applied practically for the purpose of heat dissipation [18,19,20]. Ravikumar et al. [18] found that when using the Al_2_O_3_ nanofluid as coolant of air-atomized spray cooling that it had a better enhancement of heat transfer than the base fluid for high heat flux dissipation applications. Peyghambarzadeh et al. [19] conducted an experimental study to show that the Al_2_O_3_ nanofluid, when used as car radiator coolant, has a clearly superior heat transfer enhancement compared to its base fluid. A numerically study conducted by Vajjha et al. [20] evaluated the superiority of heat transfer performance using Al_2_O_3_ and CuO nanofluids in the flat tubes of a radiator. Chinchole et al. [21] used Alumina nanofluid as emergency coolant for unclear fuel bundle because the thermal performance can be significantly enhanced. Ho et al. [22] numerically investigated the transient cooling characteristic of Al_2_O_3_–water nanofluid flow in the microchannel subjected to sudden pulsed heat flux. It is well known from the above that the base liquid combined with particles such as Al_2_O_3_ and CuO can enhance the heat dissipation of liquid cooling. As the heat cooling ability of conventional liquid cooling is limited by the heat transfer capacity of the cooling medium, such as oil and water, the application of nanofluid for electrical rotating machine cooling mission will have a big advantage relative to the base fluid, which can meet the increasing heat dissipation requirement of aircraft generators with high power density. The most studied nanoparticles are metal [21,23] and metal oxide nanoparticles [18,19,20,24,25]. Compared with the metal nanoparticles, metal oxide nanoparticles have a better nonconductivity, which means that the application of metal oxide nanoparticles into the base fluid has less influence on the electrical conversion process in BLSG. As one representative of metal oxide nanoparticles, Al_2_O_3_ nanoparticles have been widely applied in the engineering field, which has proved that the Al_2_O_3_ nanofluid possesses superior heat dissipation ability. Thus, Al_2_O_3_ nanoparticles were chosen for use in this paper.

As temperature has a big influence on the power loss and efficiency of the electrical rotating machine and the nanofluid can enhance the heat transfer rate, the application of the nanofluid could have favorable influence on the power loss and efficiency. Several studies have been devoted to the energy saving by using the nanofluid. Firouzfar et al. [26] studied the energy saving in heating ventilating and air conditioning systems. Liu et al. [27] analyzed the induction heating efficiency and thermal energy conversion ability in the roll profile electromagnetic control technology. Hassan et al. [28] studied the effect of nanofluid on thermal energy storage system using clathrate through an experimental approach. However, few studies focused on the energy saving and efficiency promotion of electrical rotating machine using nanofluid cooling. Thus, it is very significant to analyze the operating performance of the nanofluid-based cooling system (NBCS) for BLSG.

This paper is devoted to a numerical investigation on thermal performance and efficiency analysis of the proposed NBCS for a BLSG by using the Al_2_O_3_ nanofluid, whose base fluid is lubricating oil. The models of nanofluid thermophysical, heat transfer coefficients, NBCS thermal network, and NBCS efficiency are established herein. Based on these models and the arranged simulation cases, the steady-state and transient thermal performance and efficiency analysis under different Al_2_O_3_ nanoparticle volume fractions are conducted, and the results are discussed in detail. The remainder of this paper organized as follows. A summary of the idea of NBCS and mathematic models is provided in Section 2. Initial operation parameters and simulation cases for transient and steady-state thermal performance and efficiency analysis are detailed in Section 3. Simulation results and discussions are given in Section 4. Finally, the main conclusions are summarized in Section 5.

## 2. Idea of Nanofluid-Based Cooling System and Mathematic Models 

### 2.1. Idea of Nanofluid-Based Cooling System 

#### 2.1.1. Description of Nanofluid-Based Cooling System

The traditional heat dissipation method for BLSG requires its use with lubricating oil. However, it cannot meet the increasing heat dissipation demand of onboard BLSG with high density because the limitation of heat dissipation capacity of lubricating oil. The heat transfer capacity of nanofluid is far more than of its base fluid [18,19,20,21,22,23,24,25]. Thus, a nanofluid-based cooling system (NBCS) for the BLSG, which has high thermal dissipation demand, is proposed in this paper.

**Figure 1:**
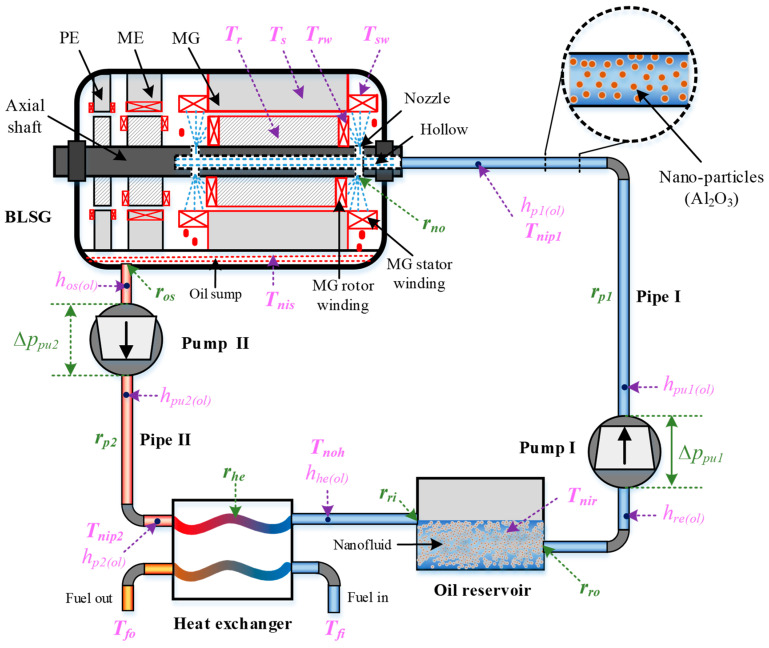
Schematic diagram of the nanofluid-based cooling system (NBCS).

The NBCS, shown in Figure 1, consists of a BLSG, two cycling pumps (pump I and II), a heat exchanger (HE), an oil reservoir, connection pipes (pipe I and II), and nanofluid cooling medium. The BLSG is composed of three cascaded different functional sub generators, which are the pilot exciter (PE), main exciter (ME), and main generator (MG). The cycling pumps function to drive the flow of nanofluid in NBCS. The heat exchanger is used to transfer the heat brought by the nanofluid flowing through the hot end of the HE to the fuel flowing through the cold end. The oil reservoir is used to store nanofluid. The nanofluid in the NBCS is Al_2_O_3_ lubricating oil nanofluid which is formed by adding Al_2_O_3_ nanoparticles into the base fluid (lubricating oil).

The mechanism of the NBCS is described as follows. Waste heat is generated during the operation of BLSG when the temperature increases; the locations with temperature increase are marked in red in BLSG in Figure 1. Because the heat losses in PE and ME are small and those in MG are big, the temperature increases caused by heat losses in PE and ME are low and those in MG are high. Thus, the primary cooled object is MG, especially the MG stator and rotor windings, which would dramatically influence the normal operation if they are not kept in a reasonable range. The MG stator and rotor are not directly cooled by the spray nanofluid. The heat dissipations of the MG stator and rotor occur through heat conduction with the MG stator and rotor winding, respectively. The cold nanofluid stored in the oil reservoir is pumped via pump I into pipe I, and flows into the hollow of BLSG after it crosses pipe I. Then, it is sprayed to the MG stator and rotor windings in BLSG via the nozzles at the ends of the MG shaft. The nanofluid becomes hot after cooling the MG stator and rotor winding. Meanwhile, it drops from the MG stator and rotor winding surface to the bottom of BLSG under gravity and gathers in the oil sump. Pump II is used to absorb the heated nanofluid out of the oil sump to keep a normal operation of the BLSG. Before returning to the oil reservoir, the heat nanofluid is delivered through the heat exchanger, where the heat in nanofluid is transfer to the cooling fuel. Finally, the cooled nanofluid travels back to the oil reservoir papering for the next cooling cycle.

#### 2.1.2. Cooling Object and Its Thermal Generation Performance 

**Figure 2:**
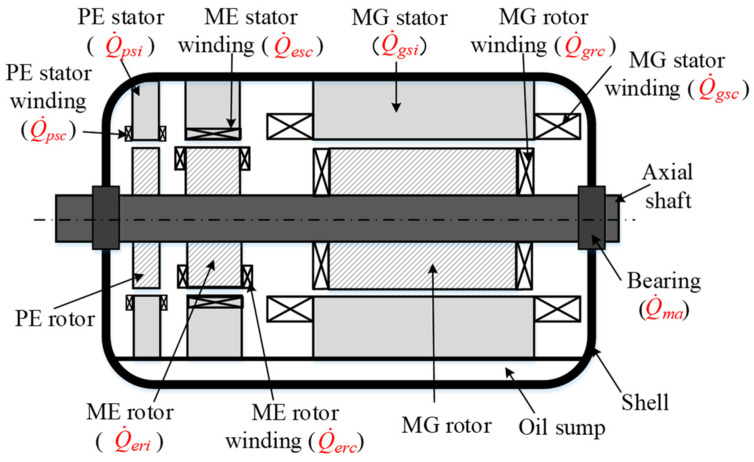
The locations of power losses in BLSG.

The onboard BLSG provides a nominal 65 kW output with a DC voltage of 270 V. The power losses generated in the working process of BLSG are mainly divided into three categories: copper loss, iron loss, and machine loss [29]. Locations of the various power losses are given in Figure 2. The copper losses in BLSG include PE stator copper loss ${\dot{Q}}_{psc}$, ME stator copper loss ${\dot{Q}}_{esc}$, ME rotor copper loss ${\dot{Q}}_{erc}$, MG stator copper loss ${\dot{Q}}_{gsc}$, and MG rotor copper loss ${\dot{Q}}_{grc}$. It has to be noted that the copper losses are significantly affected by the temperature of winding. The iron losses in BLSG includes MG, ME, and PE iron loss, which are marked as ${\dot{Q}}_{gsi}$, ${\dot{Q}}_{eri}$ and ${\dot{Q}}_{psi}$, respectively. Machine loss ${\dot{Q}}_{ma}$ in BLSG is a consequence of all kinds of frication losses.

The power losses are the heat source of BLSG, thus their calculation is significant to the thermal performance and efficiency analysis. According to the knowledge of electrical circuits and previous research [30,31,32,33], the equations to calculate the power losses can be obtained. The efficiency of the BLSG, $\eta_{G}$, is determined by its output power, $P_{o}$, and total power loss, ${\dot{Q}}_{su}$, which is the summation of copper losses, iron losses, and mechanical losses in BLSG, thus it can be expressed as Equation (1). The calculation methods of copper loss, iron loss, and mechanical loss are given in Appendix A (a).

(1) $$\eta_{G}=\frac{P_{o}}{P_{o}+{\dot{Q}}_{su}}$$

### 2.2. Mathematic Model of Nanofluid-Based Cooling Fluid

The mathematic models of the NBCS established in this section are used to support the thermal performance analysis. Specifically, the models function to analyze the thermal state of the NBCS, and are provided in Section 2.2.1. The models to obtain the flow state of nanofluid are given in Section 2.2.2. The models of the key parameters that impact the thermal performance dramatically are established in Section 2.2.3. The models in Section 2.2.3 are used to support the calculation of models in Section 2.2.1 and Section 2.2.2. Combining all these models, the thermal performance analysis of NBCS can be conducted.

#### 2.2.1. Models of Thermal State of Nanofluid-Based Cooling System

In order to conduct the thermal performance analysis of NBCS, the temperature model of each module in NBCS is established. Besides, the heat dissipation model of BLSG is also given to demonstrate the heat dissipation capacity of NBCS. It has to be noted that the temperature of key locations in NBCS are given in Figure 1. In the modeling of thermal state of nanofluid-based cooling system, the lumped parameters method is adopted; each module in NBCS is treated as several nodes. The energy balance equations are used to describe the energy changes of nodes. Besides, the changes of kinetic energy and potential energy in nanofluid nodes are ignored in the modeling process.

(a) Temperature model of nanofluid cooling windings in BLSG

The BLSG is cooled to make sure that the temperature of MG stator and rotor windings, which are the most important parts in BLSG, are in a suitable range, thus the temperature of the MG stator and rotor windings is the most important issue for the thermal design. In order to demonstrate the effect of the nanofluid on the temperature dynamics of the MG stator and rotor windings, their energy balance models are given in Equation (2). In the models, the MG stator winding, rotor windings, and nanofluid in BLSG are treated as individual nodes, and the energy change of the nodes occur through heat convective, heat conduction, input power, and enthalpy changes caused by flowing-in and -out of the node. The other energy balance equations to obtain the temperature changes of the other nodes in BLSG are given in Appendix A (a). Besides, the convective heat transfer coefficient $\alpha_{w}$ is applied for both MG stator and rotor winding because they are equal in value. The derivative process is given in Appendix A (e).

(2) $$\left\{ \begin{array}{l} \begin{array}{c} c_{sw}m_{sw}{\dot{T}}_{sw}=\alpha_{w}A_{sw}\left(T_{nip1}-T_{sw}\right)+\frac{\lambda_{s}}{\delta_{s}}A_{s}\left(T_{s}-T_{sw}\right)+{\dot{Q}}_{gsc}\\ c_{rw}m_{rw}{\dot{T}}_{rw}=\alpha_{w}A_{rw}\left(T_{nip1}-T_{rw}\right)+\frac{\lambda_{r}}{\delta_{r}}A_{r}\left(T_{r}-T_{rw}\right)+{\dot{Q}}_{grc} \end{array}\\ c_{nf}m_{nis}{\dot{T}}_{nis}=G_{nf}h_{pe1\left(ol\right)}-G_{nf}h_{os\left(ol\right)}+\alpha_{w}A_{rw}\left(T_{nip1}-T_{rw}\right)+\alpha_{w}A_{sw}\left(T_{nip1}-T_{sw}\right) \end{array}\right.$$

where, c, m, T, and $\dot{T}$ are the heat specific capacity, mass, temperature, and temperature gradient, respectively; the subscripts sw, rw, and nf represent the MG stator winding, MG rotor winding, and nanofluid, respectively; $\alpha_{w}$ is the convective heat transfer coefficient between nanofluid and windings (MG stator and rotor winding); $A_{sw}$ is the heat transfer area between the nanofluid and MG stator winding; $T_{nip1}$ is the temperature of nanofluid in pipe I; $A_{rw}$ is the heat transfer area between nanofluid and MG rotor winding; $\lambda_{s}$ and $\delta_{s}$ are the equivalent conductivity coefficient and thicknesses of air and insulation material in MG stator slots, respectively; $\lambda_{r}$ and $\delta_{r}$ are the equivalent conductivity coefficient and thicknesses of air and insulation material in MG rotor slots, respectively; $A_{s}$ and $A_{r}$ are the interior slot areas of MG stator and rotor, respectively; $T_{s}$ and $T_{r}$ are the temperature of the MG stator and rotor, respectively; ${\dot{T}}_{nis}$ is the temperature of nanofluid in oil sump; $G_{nf}$ is the mass flow of nanofluid in the cooling loop; and $h_{pe1\left(ol\right)}$ and $h_{os\left(ol\right)}$ are the enthalpies of nanofluid flow out of pipe I and oil sump, respectively.

The power losses ${\dot{Q}}_{gsc}$ and ${\dot{Q}}_{grc}$ are the heat sources of the MG stator and rotor windings, respectively. The MG stator winding is a three-phase one, while the MG rotor winding is single-phase, according to the calculation equation of copper loss given in Appendix A (a), the expressions of ${\dot{Q}}_{gsc}$ and ${\dot{Q}}_{grc}$ are given as

(3) $$\left\{ \begin{array}{l} {\dot{Q}}_{gsc}=3I_{sw}^{2}\left[1+k_{R}(T_{sw}-20)\right]{R^{\prime }}_{sw}\\ {\dot{Q}}_{grc}=I_{rw}^{2}\left[1+k_{R}(T_{rw}-20)\right]{R^{\prime }}_{rw} \end{array}\right.$$

where $I_{sw}$ and $I_{rw}$ are root mean square of the MG stator and rotor winding currents, respectively; $k_{R}$ is the temperature coefficient of winding; and ${R^{\prime }}_{sw}$ and ${R^{\prime }}_{sw}$ are the effective electrical resistance of the MG stator and rotor winding respectively.

(b) Temperature model of nanofluid in the pipe

The temperature models of the nanofluid in pipes I and II have the same form. Taking pipe I as an example, the equation to describe the temperature dynamic of nanofluid in pipe I is given as

(4) $$c_{nf}m_{nip1}{\dot{T}}_{nip1}=\alpha_{pe}A_{pe}\left(T_{pe1}-T_{nip1}\right)+G_{nf}h_{pu1\left(ol\right)}-G_{nf}h_{pe1\left(ol\right)}$$

where $m_{nip1}$ is the mass of nanofluid in pipe I, $\alpha_{pe}$ is the convective heat transfer coefficient between the nanofluid and the pipe, $A_{pe}$ is the contact area between the nanofluid and the pipe, $T_{pe1}$ is the temperature of pipe I, and $h_{pu1\left(ol\right)}$ is the enthalpy of nanofluid flow out of pump 1.

(c) Temperature model of heat exchanger

The model to describe the temperature of fluid flow in HE is given as

(5) $$\left\{ \begin{array}{l} T_{noh}=T_{nip2}-\eta_{he}\left(T_{nip2}-T_{HE}\right)\\ T_{fo}=T_{fi}-\eta_{ce}\left(T_{fi}-T_{HE}\right)\\ m_{HE}c_{HE}{\dot{T}}_{HE}=\eta_{he}G_{nf}c_{nf}\left(T_{nip2}-T_{HE}\right)+\eta_{he}G_{f}c_{f}\left(T_{fi}-T_{HE}\right) \end{array}\right.$$

where $T_{noh}$ is the temperature of nanofluid flow out of the hot end of HE; $T_{nip2}$ is the temperature of nanofluid in pipe II, which is also the temperature of nanofluid flow in the hot end of HE; $\eta_{he}$ and $\eta_{ce}$ are the heat transfer efficiency of the hot end and cold end in the HE, respectively; $T_{HE}$ is the temperature of HE; and $T_{fi}$ and $T_{fo}$ are the temperature of fuel flow-in and -out of the cold end of HE, respectively. $m_{HX}$ and $c_{HX}$ are the mass and specific heat capacity of HE, respectively, and $T_{HE}$ is the temperature gradient of HE.

(d) Temperature model of nanofluid in reservoir

The temperature dynamic of nanofluid in reservoir can be derived by

(6) $$c_{nf}m_{nir}{\dot{T}}_{nir}=\alpha_{re}A_{re}\left(T_{re}-T_{nir}\right)+G_{nf}h_{he\left(ol\right)}-G_{nf}h_{re\left(ol\right)}$$

where $m_{nir}$ is the mass of nanofluid in reservoir, $T_{nir}$ is the temperature of nanofluid in reservoir, ${\dot{T}}_{nir}$ is the temperature gradient of $T_{nir}$, $\alpha_{re}$ is the convective heat transfer coefficient between nanofluid and reservoir, $A_{re}$ is the contact area between nanofluid and reservoir, $T_{re}$ is the temperature of reservoir, and $h_{re\left(ol\right)}$ is the enthalpy of nanofluid flow out of reservoir.

(e) Temperature model of pump

As for the modeling of pump, the input power is considered to completely covert into the enthalpy change of nanofluid flowing through it. Thus, taking pump I as an example, the model of pump I is given as

(7) $$G_{nf}\left(h_{re\left(ol\right)}-h_{pu1\left(ol\right)}\right)=P_{in,pu1}$$

where $h_{pu1\left(ol\right)}$ is the enthalpy of nanofluid flow out of pump I and $P_{in,pu1}$ is the input power of pump I.

(f) Heat dissipation models of BLSG

The most important cooling object in NBCS is the BLSG, thus the heat dissipation of BLSG is a worthy concern. The heat in the BLSG is dissipated in two ways, which are nanofluid cooling and heat leakage. The equations to describe the two dissipation heat are given as

(8) $${\dot{Q}}_{nf}=G_{nf}c_{nf}\left(T_{nis}-T_{nip1}\right)$$

(9) $${\dot{Q}}_{le}=\alpha_{a}A_{sh}\left(T_{sh}-T_{a}\right)+\frac{\lambda_{st}}{\delta_{st}}A_{st}\left(T_{sh}-T_{st}\right)$$

where ${\dot{Q}}_{nf}$ and ${\dot{Q}}_{le}$ are the nanofluid dissipation heat and leakage heat, respectively; $T_{sh}$ is the temperature of BLSG shell; $T_{a}$ and $T_{st}$ are the temperature of the ambient and installing structures, respectively; $\alpha_{a}$ is the convective heat transfer coefficient between shell of BLSG and ambient; $A_{sh}$ is the surface area of BLSG; $\lambda_{st}$ is the equivalent conductivity coefficient of installation material; $\delta_{st}$ is the equivalent thickness of air and installation material; and $A_{st}$ is the contact area of installation structure.

#### 2.2.2. Models of Flow State of Nanofluid-Based Cooling System

The flow state models in this section are used to obtain the pressure drops, flow resistances, and power loss in the loop of NBCS. The loop of NBCS can be divided into two sections according to the flow state of nanofluid. The first section is composed of oil reservoir outlet, pump I, pipe I, and BLSG nozzles. It functions to deliver the nanofluid from the reservoir to the internal space of BLSG. The second section includes oil sump, pump II, pipe II, heat exchange hot end, and reservoir inlet, and is used to extract the redundant nanofluid from the oil sump to the reservoir. Pumps I and II, in the two sections, are used for boosting the pressure of nanofluid. Other components that can be characterized by the flow resistance, on the other hand, reduce the pressure. Besides, the nanofluid mass flow in the first section is equal to that of the second section. The pressure heads of the pumps and the resistances of modules in the NBCS are symbolized in green and shown in Figure 1. According to the flow equation [34], the flow characteristics of the two sections can be described as Equations (10) and (11), respectively.

(10) $$\Delta p_{pu1}=\left(r_{ro}+r_{pe1}+r_{nos}\right)G_{nf}^{2}$$

(11) $$\Delta p_{pu2}=\left(r_{os}+r_{pe2}+r_{he}+r_{ri}\right)G_{nf}^{2}$$

where $\Delta p_{pu2}$ and $\Delta p_{pu2}$ are the pressure heads of pump I and II, respectively; $r_{ro}$, $r_{pe1}$, and $r_{nos}$ are the flow resistances of reservoir outlet, pipe I, and all nozzles in the first section, respectively; and $r_{os}$, $r_{pe2}$, $r_{he}$, and $r_{ri}$ are the flow resistances of oil sump, pipe II, hot end of HE, and reservoir inlet in the second section.

All the flow resistances in the NBCS are composed of two kinds of flow resistances, which are local flow resistance and friction flow resistance [35]. The expressions of local flow resistance $r_{l}$ and friction loss flow resistance $r_{f}$ are given in Equations (12) and (13), as follows.

(12) $$r_{l}=\frac{k_{l}}{2\rho_{nf}A_{du}^{2}}$$

(13) $$r_{f}=\frac{k_{f}l_{du}}{2\rho_{nf}A_{du}^{2}d_{du}}$$

where $r_{l}$ and $r_{f}$ are the local flow resistance and friction loss flow resistance, respectively; $k_{l}$ and $k_{f}$ are the coefficients of local flow resistance and friction loss flow resistance, respectively; $A_{du}$ is the cross-sectional area of duct; $l_{du}$ is the length of duct; and $d_{du}$ is the diameter of duct.

Note that the nanofluid in the BLSG is sprayed via the same four nozzles distributed at both ends of MG shaft. Thus, the flow resistance of the nozzles $r_{nos}$ is equal to eight flow resistances in parallel, which can be described as

(14) $$r_{nos}=\frac{r_{no}}{8}$$

where $r_{no}$ is the flow resistance of a single nozzle.

The input power of the pumps is determined by their pressure head and mass flow, thus the equations of pump I and II input power can be described as

(15) $$P_{in,pu1}=\frac{p_{pu1}G_{nf}}{\eta_{pu1}\rho_{nf}}$$

(16) $$P_{in,pu2}=\frac{p_{pu2}G_{nf}}{\eta_{pu2}\rho_{nf}}$$

where $P_{in,pu1}$ and $P_{in,pu2}$ are the input power of pumps I and II, respectively, and $\eta_{pu1}$ and $\eta_{pu2}$ are the efficiency of pumps I and II respectively.

In the NBCS, pump I and II consume much of the electrical power, thus the input power of pumps I and II should be considered in the efficiency calculation of the NBCS. Thus, the efficiency of NBCS $\eta_{sy}$ is given by

(17) $$\eta_{sy}=\frac{P_{o}}{P_{o}+{\dot{Q}}_{su}+P_{in,pu1}+P_{in,pu2}}$$

#### 2.2.3. Models of Key Parameters Impact on Thermal Transfer

As described above, the thermophysical parameters of nanofluid, such as density and specific heat capacity, and the convective heat transfer coefficients are the key input parameters of the models in Section 2.2.1, which impact the thermal performance of NBCS dramatically. Besides, the thermophysical parameters of the nanofluid are also key input parameters of the models in Section 2.2.2, which have a big influence on the models of flow state. Thus, the models of thermophysical parameters and convective heat transfer coefficients are established in this section to support the thermal and flow state analysis of NBCS.

(a) Models of nanofluid thermophysical parameters

It is well known that the nanoparticle volume fraction (NVF) of a nanofluid has tremendous influence on the thermophysical parameters of that nanofluid. The involved thermophysical parameters of the nanofluid in this paper are density, specific heat capacity, thermal conductivity, and viscosity. The models of density under different NVF is given as [36]

(18) $$\rho_{nf}=\phi_{p}\rho_{p}+\left(1-\phi_{p}\right)\rho_{oi}$$

where $\phi_{p}$ is the nanofluid volume fraction and $\rho_{p}$ and $\rho_{oi}$ are the density of nanoparticle and lubricating oil, respectively. The model of specific heat capacity is given by [37]

(19) $$c_{nf}=\frac{\phi_{p}\rho_{p}c_{p}+\left(1-\phi_{p}\right)\rho_{oi}c_{oi}}{\rho_{nf}}$$

where $c_{p}$ and $c_{oi}$ are the specific heat capacity of nanoparticle and lubricating oil, respectively. The model of thermal conductivity is given as [38]

(20) $$\lambda_{nf}=\frac{\lambda_{p}+2\lambda_{oi}+2\phi_{p}\left(\lambda_{p}-\lambda_{oi}\right)}{\lambda_{p}+2\lambda_{oi}-\phi_{p}\left(\lambda_{p}-\lambda_{oi}\right)}\lambda_{oi}$$

where $\lambda_{p}$ and $\lambda_{oi}$ are the thermal conductivity of nanoparticle and lubricating oil, respectively. The model of viscosity is given by [39]

(21) $$\mu_{nf}=\mu_{oi}\left(1+2.5\phi \right)$$

where $\mu_{oi}$ is the viscosity of the nanoparticle and lubricating oil, respectively.

(b) Convective heat transfer coefficient between the sprayed nanofluid and MG stator and rotor windings

In the modeling of the convective heat transfer coefficient between the sprayed nanofluid and MG stator and rotor windings, several assumptions are given: (1) the heat transfer is considered as single-phase cooling and (2) the effect of gravity and inclination in the spray cooling is neglected. According to Rybicki’s study [40], the convective heat transfer coefficient of nanofluid spray cooling $\alpha_{w}$ is given as follows

(22) $$\alpha_{w}=\frac{Nu_{w}\lambda_{nf}}{d_{32}}$$

where $d_{32}$ is the Sauter mean diameter of nanofluid and $Nu_{w}$ is the Nusselt number, which can be calculated by [40,41]

(23) $$Nu_{w}=4.70Re_{w}^{0.61}Pr_{nf}^{0.32}$$

where $Re_{w}$ and $Pr_{nf}$ are the Reynolds number and Prandtl number, respectively, they are defined as Equations (24) and (25), respectively

(24) $$Re_{w}=\frac{\rho_{nf}d_{32}V}{\mu_{nf}}$$

(25) $$Pr_{nf}=\frac{\mu_{nf}c_{nf}}{\lambda_{nf}}$$

where V is the volumetric flux which can be calculated by

(26) $$V=\frac{q_{nf}}{A_{sp}}$$

where $q_{nf}$ is the volumetric flow rate (VFR) of the nanofluid and is determined by the nanofluid mass flow $G_{nf}$ and density $\rho_{nf}$, which are expressed in Equation (27).

(27) $$q_{nf}=\frac{G_{nf}}{\rho_{nf}}$$

Based on Equations (22)–(27), the convective heat transfer coefficient between the sprayed nanofluid and the windings in the BLSG is given as

(28) $$\alpha_{w}=4.70\frac{G_{nf}^{0.61}c_{nf}^{0.32}\lambda_{nf}^{0.68}}{\rho_{nf}^{0.39}A_{sp}^{0.61}\mu_{nf}^{0.29}d_{32}^{0.39}}$$

(c) Convective heat transfer coefficient between nanofluid and pipe

According to the definition of Nusselt number, the convective heat transfer coefficient between the pipe and nanofluid in it $\alpha_{pe}$ can be calculated by

(29) $$\alpha_{pe}=\frac{Nu_{pe}*k_{nf}}{d_{pe}}$$

where $Nu_{pe}$ is the Nusselt number of nanofluid in pipes. According to Xuan et al. [42], the correlation between the $Nu_{pe}$ under laminar flow and turbulent flow is given as Equation (30).

(30) Nu=0.0059(1+7.6286ϕ0.6886Pepe0.001)Repe0.9238Prnf0.4(turbulent flow)Nu=0.4328(1+11.285ϕ0.754Pepe0.218)Repe0.333Prnf0.4(laminar flow)

where $Re_{pe}$ and $Pe_{pe}$ are the Reynolds number and Peclet number, respectively, their expression is given as follows

(31) $$Re_{pe}=\frac{u_{pe}d_{pe}\rho_{nf}}{\mu_{nf}}$$

(32) $$Pe_{pe}=\frac{u_{pe}d_{p}}{\sigma_{nf}}$$

where $u_{pe}$ is the flow velocity of nanofluid in pipes and $\sigma_{nf}$ is the thermal diffusivity of the nanofluid. The expressions of $u_{pe}$ and $\sigma_{nf}$ are given as

(33) $$u_{pe}=\frac{4q_{nf}}{\pi d_{nf}^{2}}$$

(34) $$\sigma_{nf}=\frac{\lambda_{nf}}{\rho_{nf}c_{nf}}$$

Substituting Equations (30)–(34) into Equation (29), the convective heat transfer coefficient between the pipe and nanofluid ($\alpha_{pe}$) can be obtained.

### 2.3. The Calculation Processes

**Figure 3:**
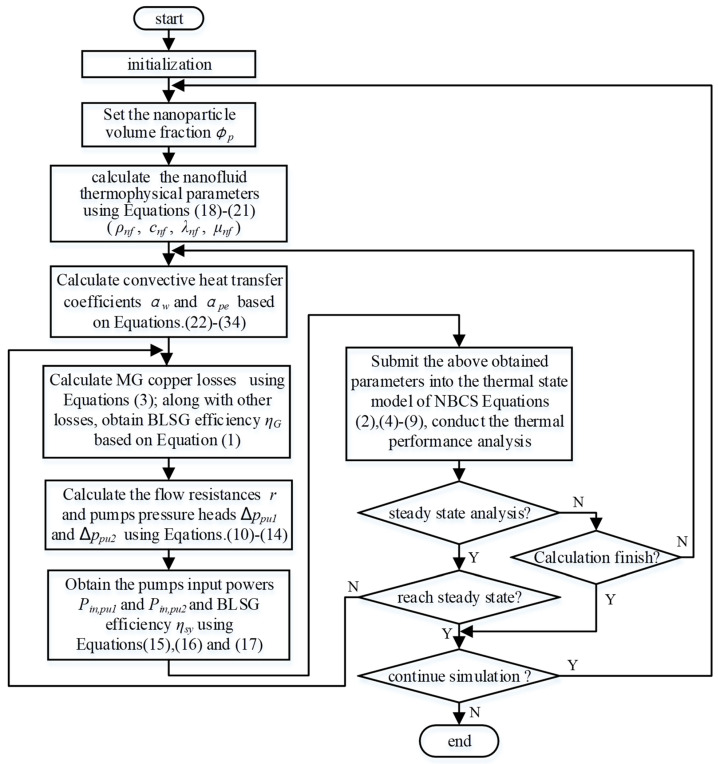
The flowchart of the calculation procedure.

The mathematical model of NBCS in Section 2.3 is programmed using MATLAB. The model calculation is conducted in steps, as shown in Figure 3, and is described in detail in the following.

(1) In the beginning, the calculation program is initialized where basic simulation parameters, including the NBCS physical parameters and initial work condition, are inputs. Additionally, the simulation step size and calculation time are set.
(2) The NVF $\phi_{p}$ is set to calculate the nanofluid thermophysical properties using Equations (18)–(21).
(3) According to the initial work condition, the convective heat transfer coefficients $\alpha_{w}$ and $\alpha_{pe}$ can be obtained based on Equations (22)–(34).
(4) In order to obtain the heat generation in Equation (2), the copper losses of the MG stator and rotor windings are calculated using Equation (3). After all power losses in BLSG are obtained, the efficiency of BLSG $\eta_{G}$ is calculated by using Equation (1).
(5) All flow resistances in the NBCS and pump pressure heads $p_{pu1}$ and $p_{pu2}$ are calculated using Equations (10)–(14).
(6) The pump input powers $P_{in,pu1}$ and $P_{in,pu2}$ can be calculated using Equations (15) and (16), respectively. Based on the power losses of BLSG and input power of the pumps obtained above, the efficiency of the NBCS $\eta_{sy}$ is calculated by Equation (17).
(7) The parameters obtained above are all submitted into the thermal state model of NBCS to conduct the thermal performance analysis based on Equations (2) and (4)–(9).
(8) Lastly, some judgments need to be made. The first judgment is whether the thermal performance analysis is a steady-state analysis. If yes, it means that the simulation is used for steady-state thermal performance analysis. Then, the second judgment is whether the simulation reaches the steady-state. If the second judgment is no, the calculation will jump to step (4). If the second judgment is yes, the calculation step continues to the third judgment, which is whether the calculation will continue. If the third judgment is no, the simulation ends or it will jump to step (2). If the first judgment is no, the simulation is transient for thermal performance analysis. In the following, the fourth judgment, which is whether the calculation is finished need to be made, if yes, the simulation will jump to the third judgment, if not, the simulation jump to step (3).

## 3. Parameter Determinations and Simulation Cases

### 3.1. Physical Parameters and Initial Operation Condition

In this section, some physical parameters and initial operation conditions of the NBCS are given. Specifically, the basic physical parameters of the BLSG, which is the most important module in NBCS, are given in the left side of Table 1. To demonstrate the effect of nanofluid, and to make comparison of thermal performance between NBCS and base fluid cooling system, the initial condition of base fluid cooling system is set as the initial condition of this study. Key parameters of the initial condition are listed in the right side of Table 1. Besides, the thermophysical parameters of Al_2_O_3_ particles [20] and lubricating oil [43] are listed in Table 2 to calculate the thermophysical properties of the Al_2_O_3_ lubricating oil nanofluid.

### 3.2. Simulation Cases Arrangement 

The focus of this paper is to investigate the advantages of using Al_2_O_3_ lubricating oil nanofluid as the cooling medium on the thermal performance and efficiency of the NBCS. Specifically, the transient and steady-state thermal performance of NBCS is studied. The power losses and efficiency of the NBCS are analyzed as well. The simulation cases for transient, steady-state thermal performances and efficiency analysis are described in Section 3.2.1, Section 3.2.2, and Section 3.2.3, respectively.

#### 3.2.1. Simulation Cases Arrangement for Steady-State Thermal Performance Analysis

It is well known from the studies conducted by many researchers [19,20,44] that the NVF has a big influence on the thermal performance of cooling system. Thus, the steady-state thermal performance under different NVFs should be investigated to demonstrate the nanofluid cooling effect. In this paper, the cases of the Al_2_O_3_ NVF, from 0% to 10% with 1% increment, are arranged to study the steady-state thermal performance, which are listed in Table 3. The reason for the use of this range of NVF is that the nanofluid within the selected range has been extensively utilized [45]. Note that the other parameters of the operating condition, especially the nanofluid VFR $q_{nf}$ remain as the initial condition, which is listed in Table 1.

#### 3.2.2. Simulation Cases Arrangement for Transient Thermal Performance Analysis

The thermal performance of the NBCS is mainly determined by heat generation and dissipation. In the NBCS, the output power $P_{o}$ is one of the most important parameters to determine the power losses that are converted into the waste heat. The nanofluid VFR $q_{nf}$ determines the heat transfer between the nanofluid and BLSG, which determines the heat dissipation process. Thus, the BLSG output power $P_{o}$ and nanofluid VFR $q_{nf}$ affect the heat thermal performance dramatically. In this paper, the effect of the BLSG output power $P_{o}$ and nanofluid VFR $q_{nf}$ are analyzed by changing the corresponding parameter. The arranged simulation cases are listed in Table 4. A 20% step reduction in output, $P_{o}$, in case I lead to a reduction in the heat generation rate, which lead to a decrease in the BLSG temperature. In Case II, a 20% step reduction of nanofluid VFR ($q_{nf}$) resulted in a heat dissipation capacity decrease in the nanofluid, which eventually led to BLSG temperature increase. Thus, the two cases with different NVFs are set to illustrate the nanofluid cooling effect on NBCS transient thermal performance when temperature increase and decrease. The prescheduled step disturbance took place at 300 s in each case when the system had reached a steady-state under the initial state. Both cases were simulated under the NVF range between 0% and 10%.

#### 3.2.3. Simulation Cases Arrangement for Power Loss and Efficiency Analysis

As analyzed above, the NVF will affect the thermal performance of the NBCS, and thus influence the power losses. Besides, the input power of pumps in the NBCS is also influenced by the NVF because the thermophysical properties, such as nanofluid density and viscosity, vary significantly when the NVF changes, which will lead to a change in the input power of the pumps. In order to demonstrate the effect of the NVF on the power losses and efficiency of NBCS, cases under different NVFs have been arranged. Because the analysis of power losses and efficiency is under steady-state, the cases arranged for power losses and efficiency analysis are the same as the cases in Table 3.

## 4. Results and Discussions

The thermal performance and efficiency of the NBCS under base fluid cooling are investigated firstly to form a reference to the performance under different NVF cooling conditions. Some key parameters that can represent the thermal performance and efficiency of NBCS are calculated under base fluid cooling and initial working condition in Table 1. They are displayed in Table 5.

To clearly demonstrate the changes of thermal performance and efficiency of NBCS under different NVF, the key parameters, which can represent the thermal performance and efficiency of NBCS, are normalized to their reference values listed in Table 5. The normalized function ψ(x) is defined as Equation (35), where ψ is the normalized function, x is one of the key parameters, $x^{0}$ is the parameter under base fluid cooling condition, and $x^{\phi }$ is the parameter under nanofluid cooling condition when NVF is ϕ. Besides, the function φ(x), defined in Equation (36), is used to present the changes of parameter under the same NVF, where $x^{\phi ,0}$ is the initial value of the parameter when NVF is ϕ. Note that $x^{\phi }$ will vary during the working process.

(35) $$\psi \left(x\right)=\frac{x^{\phi }}{x^{0}}$$

(36) $$\varphi \left(x\right)=\frac{x^{\phi }}{x^{\phi ,0}}$$

### 4.1. Effect of Thermal Properties of Nanofluid

**Figure 4:**
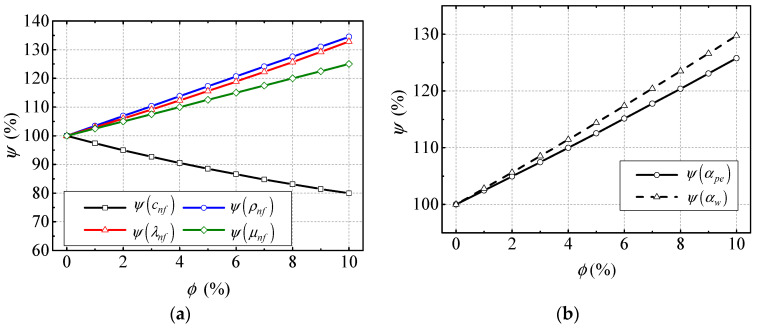
Normalized thermophysical property and convective heat transfer coefficients of nanofluid under different volume fraction. (**a**) Normalized thermophysical property. (**b**) Normalized convective heat transfer coefficients.

The typical thermophysical parameters and heat transfer coefficients are shown in Figure 4. Specifically, the normalized thermophysical parameters and heat transfer coefficients are shown in Figure 4a,b, respectively. The results in Figure 4a show that the normalized density, thermal conductively, and viscosity, symbolized as $\psi \left(\rho_{nf}\right)$, $\psi \left(\lambda_{nf}\right)$ and $\psi \left(\mu_{nf}\right)$ respectively, all linearly increase with the increasing of NVF in nanofluid, while the growth rates are different. The normalized specific heat $\psi \left(c_{nf}\right)$ shows a reverse trend. The results in Figure 4a indicate that the NVF has a big influence on the thermophysical parameters of the nanofluid. Figure 4b shows that the normalized convective heat transfer coefficients between the sprayed nanofluid and MG stator and rotor windings $\psi \left(\alpha_{w}\right)$ increase with increasing NVF. The normalized convective heat transfer coefficient between the nanofluid and pipe has the same variation trend. Their increments are 63% and 58%, respectively, when the NVF is 10%. Therefore, it can be deduced that the increase in NVF enhances the heat transfer between the nanofluid and windings and pipes.

### 4.2. Effect of Nanofluid on Steady-State Thermal Performance

**Figure 5:**
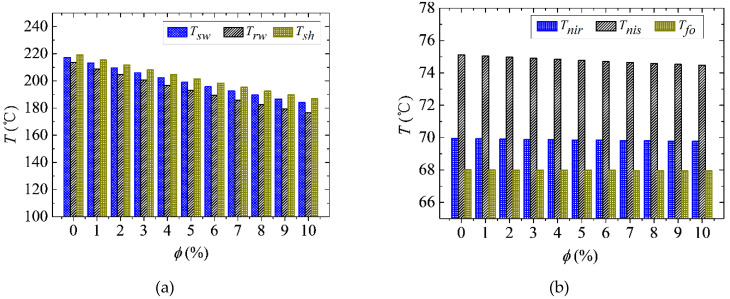

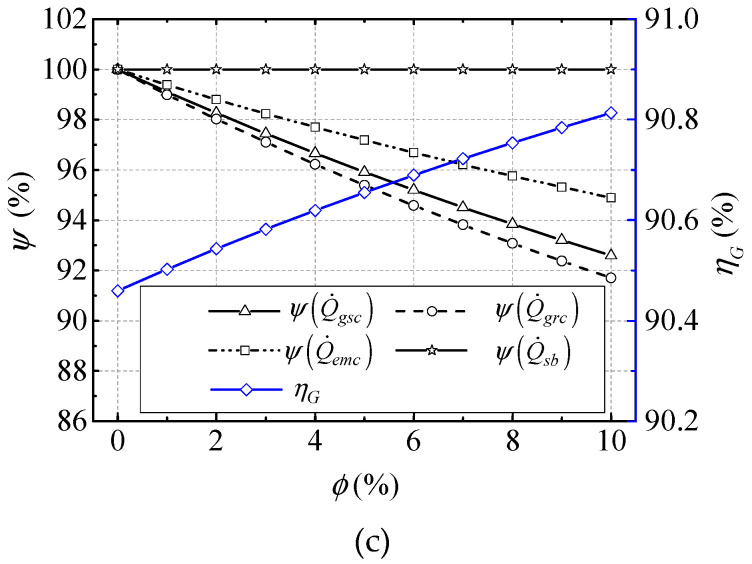
Steady-state temperature in NBCS: (**a**) MG stator and rotor winding temperature and shell temperature; (**b**) temperature of oil in tank, oil sump, and fuel in the heat exchanger cold end outlet; and (**c**) power losses and efficiency of BLSG.

In order to illustrate the steady-state thermal performance of NBCS under different Al_2_O_3_ NVFs, some key parameters are shown in Figure 5. Specifically, the BLSG MG stator and rotor winding temperatures, $T_{sw}$ and $T_{rw}$, respectively, and shell temperature, $T_{sh}$, are presented in Figure 5a; while the temperature of nanofluid in BLSG oil sump and oil reservoir, $T_{nis}$ and $T_{nir}$, respectively, and heat exchanger cold end outlet fuel temperature, $T_{cof}$, are illustrated in Figure 5b, and the normalized copper losses and efficiency of BLSG are shown in Figure 5c. In order to facilitate to analysis, the summation of copper losses in PE and ME is remarked as ${\dot{Q}}_{emc}$ because it is much less than that in MG. The iron loss and machine loss in BLSG are considered to be insensitive to the temperature change and their summation is represented as ${\dot{Q}}_{su}$.

It can be found from Figure 5a that the steady-state temperature of MG stator and rotor winding, $T_{sw}$ and $T_{rw}$, respectively, and shell temperature $T_{sh}$ are 217.3 °C, 213.4 °C, and 219.3 °C, respectively, when the NVF is 0% (basic fluid). With the NVF increases, those temperatures are all decreased significantly. Their biggest temperature drops are 33.2 °C, 36.9 °C, and 32.2 °C, respectively, at 10% NVF. Because the convective heat transfer coefficient between the nanofluid and windings $\alpha_{w}$ is increased with increasing NVF, which can be found in Figure 4b, the heat transfer rate between the nanofluid and MG stator and rotor winding are also increased, which finally contributes to the MG stator and rotor winding temperature, $T_{sw}$ and $T_{rw}$, respectively, decrease with the NVF increasing. Besides, because the copper losses in MG stator and rotor winding are the heat sources of the windings, and the decreases of MG stator and rotor winding temperature, $T_{sw}$ and $T_{rw}$, respectively, can also contribute to the decline in copper losses in the MG stator and rotor winding, which can be found in Figure 5c. It is found that the normalized MG stator copper loss $\psi \left({\dot{Q}}_{gsc}\right)$, rotor copper loss $\psi \left({\dot{Q}}_{grc}\right)$, and entirety copper losses in PE and ME $\psi \left({\dot{Q}}_{emc}\right)$ are nearly linearly decreased with the increasing of Al_2_O_3_ NVF in Figure 5c. Their descend ranges are 7.4%, 8.3%, and 5.1%, respectively, when the NVF is 10%. $\psi \left({\dot{Q}}_{sb}\right)$—the summation of all kinds of power loss except the copper loss in BLSG—was found to be unchanged, which means the application of nanofluid cooling has little influence on it. Because the electrical current in stator and rotor windings remain the same when the BLSG is under the steady-state, it can be deduced from Equation (3) that the drops of copper losses are due to the temperature declines of copper windings, which can be found in Figure 5a. It is the increasing heat transfer coefficient between the nanofluid and windings that led to the temperature decline in the winding temperatures $T_{sw}$ and $T_{rw}$, which eventually resulted in the decrease in copper losses. In return, the decreasing copper losses means that the generated waste heat is decreased, which contributes to the temperature reductions. The winding temperature and copper power losses are positive feedback. Besides, the BLSG efficiency $\eta_{G}$ increases from 90.46% to 90.81% when the NVF increases from 0% to 10%. It can be deduced that the efficiency promotion is mainly contributed to the copper losses reduces. The results show that the application of nanofluid cooling can enhance the heat transfer between the nanofluid and winding, reduce the copper power loss of BLSG, and promote its efficiency. Besides, after the temperature decline, copper power losses reduce and the efficiency promotion is positive while the NVF is increasing.

In Figure 5b, the temperature of nanofluid in BLSG oil sump and reservoir, $T_{nis}$ and $T_{nir}$, respectively, and fuel temperature of heat exchanger cold end outlet, $T_{cof}$, all drop slightly with the NVF increase. The maximum decreases are 0.64 °C, 0.18 °C, and 0.06 °C, respectively, when the NVF is 10%. Because the fuel outlet temperature of the heat exchanger cold end, $T_{cof}$, is almost stable, it is indicated that the heat absorbed by the fuel decrease little. The reservoir can be regarded as both the start and end of the cooling loop, which be seen in Figure 1. Thus, the temperature of the nanofluid in oil reservoir $T_{nir}$ is determined by the heat generation and dissipation in NBCS. It can be easily obtained from Figure 5c that the total power loss in the BLSG decreased; therefore the total generated waste heat is reduced. Thus, the decrease in the heat generated in BLSG and a small drop in fuel dissipation heat in the heat exchanger cold end contribute to the small drop in temperature of the nanofluid in oil reservoir $T_{nir}$. The decline of nanofluid temperature in BLSG sump $T_{nis}$ indicates that the heat of nanofluid absorbed from the MG stator and rotor winding is reduced. It can be deduced that the huge MG stator and rotor winding temperature declines in Figure 5a as a result of the decrease in temperature difference between the sprayed nanofluid and windings, which eventually led to the decrease in heat transfer between the nanofluid and stator and rotor windings; although the heat transfer coefficient between the sprayed nanofluid and stator and rotor winding is increased with increasing NVF. Thus, the decrease in heat transfer between the sprayed nanofluid and stator and rotor windings contributes to the drop of the temperature of nanofluid in BLSG oil sump $T_{nis}$. The results in Figure 5a,b demonstrate that the applied of nanofluid can reduce not only the temperature of windings in BLSG but also the temperature of nanofluid flowing in the BLSG system. It shows that the NBCS has great advantage for heat dissipation.

During the working process of BLSG, there are two main methods to dissipate the generated heat in BLSG: heat dissipated by the nanofluid ${\dot{Q}}_{nf}$ and heat leakage through ${\dot{Q}}_{le}$. The total generated heat ${\dot{Q}}_{to}$, nanofluid heat dissipate ion ${\dot{Q}}_{nf}$, and heat leakage ${\dot{Q}}_{le}$ under different NVFs are listed in Table 6. It can be found that the total generated heat ${\dot{Q}}_{to}$ decreases with increasing NVF, which means the total power loss is decreased with the increase in Al_2_O_3_ NVF. Besides, both the nanofluid heat dissipation ${\dot{Q}}_{nf}$ and leakage heat dissipation ${\dot{Q}}_{le}$ decrease with increasing NVF. The descent rate of ${\dot{Q}}_{le}$ is bigger than that of ${\dot{Q}}_{nf}$. It can be deduced that the decline of ${\dot{Q}}_{le}$ is the reason for the decline in the temperature of shell $T_{sh}$. The decrease in nanofluid dissipation heat ${\dot{Q}}_{nf}$ mainly contributes to the drop in heat generated in the BLSG.

**Figure 6:**
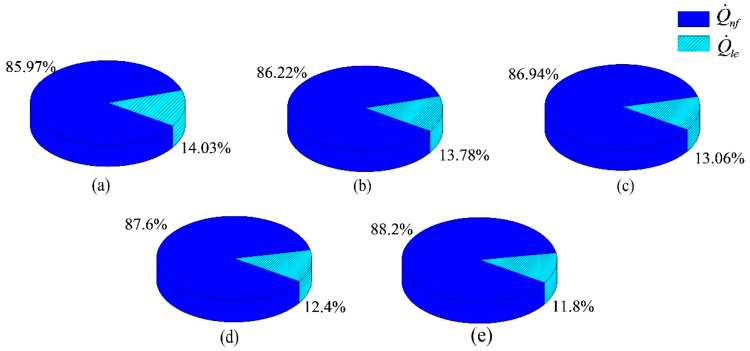
Heat dissipation proportions under different cases: (**a**) base fluid, (**b**) 1% NVF, (**c**) 4% NVF, (**d**) 7% NVF, and (**e**) 10% NVF.

In order to facilitate analysis, the proportions of heat dissipation by the two methods in the total generated heat in five chosen cases (0%, 1%, 4%, 7%, and 10%) are drawn in pipes and presented in Figure 6. Under the base fluid cooling condition (0%), the proportions of ${\dot{Q}}_{nf}$ and ${\dot{Q}}_{le}$ are 85.97% and 14.03%, respectively. With increasing NVF, the proportion of ${\dot{Q}}_{nf}$ is increased to 88.2% when the NVF is 10%, while the proportion of ${\dot{Q}}_{le}$ shrinks to 11.8%. It can be easily obtained that the decrease in ${\dot{Q}}_{le}$ proportion is due to the decline in leakage heat dissipation, ${\dot{Q}}_{le}$, in Table 6. Though ${\dot{Q}}_{nf}$ is reduced with increasing NVF, the nanofluid heat dissipation capability is improved because the heat transfer coefficient between the nanofluid and windings is increased, which contributes to the enhancement of heat transfer rate and increase in ${\dot{Q}}_{nf}$ proportion. The results indicates that cooling ability of the nanofluid in the NBCS is increase with the NVF increasing, which is beneficial to the heat dissipation of cooling object in NBCS.

### 4.3. Effect of Nanofluid on Transient Thermal Performance

**Figure 7:**
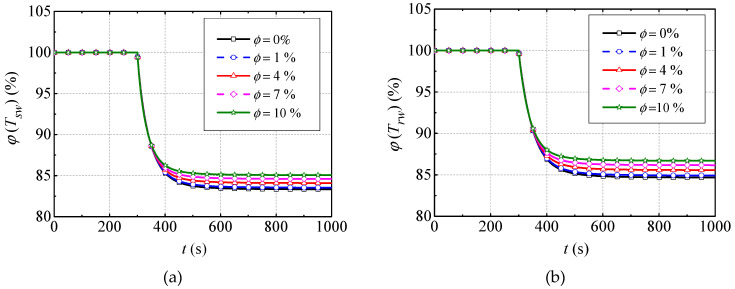

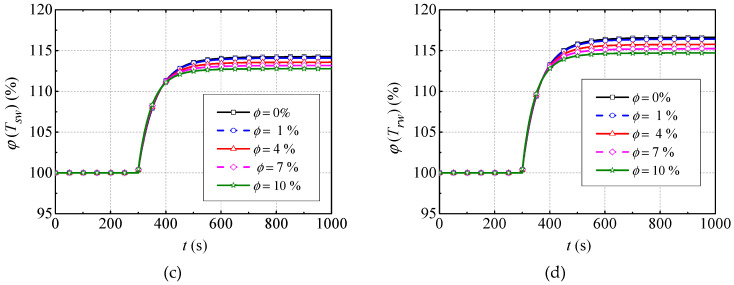
Normalized values of MG stator and rotor winding temperature responses under different cases: (**a**) MG stator temperature dynamic change under case I; (**b**) MG rotor temperature dynamic change under case I; (**c**) MG stator temperature dynamic change under case II; and (**d**) MG rotor temperature dynamic change under case II.

Based on the simulation cases for transient thermal performance analysis in Table 4, the dynamic simulations are conducted and the results are presented in Figure 7. Specifically, the normalized values $\varphi \left(T_{sw}\right)$ and $\varphi \left(T_{rw}\right)$ of the MG stator and rotor winding temperature under case I are shown in Figure 7a,b, respectively. The $\varphi \left(T_{sw}\right)$ and $\varphi \left(T_{rw}\right)$ of case II are shown in Figure 7c,d, respectively. In order to analyze the transient thermal properties of BLSG conveniently, the settling time τ(1% criterion) and change ratios of normalized temperature ξ (between the final value and its initial steady-state value) under the two cases are listed in Table 7.

It can be easy obtained that all the normalized MG stator and rotor winding temperatures ($\varphi \left(T_{sw}\right)$ and $\varphi \left(T_{rw}\right)$) under different NVF experience exponential drop with the BLSG output power $P_{o}$, decreasing by 20% in case I in Figure 7a,b, while they have exponential increases in case II with the nanofluid VFR $q_{nf}$ reducing by 20% in Figure 7c,d. It can be easily deduced that the 20% reduction in $P_{o}$ causes the decline of heat generation rate, which contributes to decrease of temperature in Figure 7a, b. The 20% reduction in $q_{nf}$ leads to the decrease in heat dissipation rate, which contributes to the increase in temperature in Figure 7c,d.

Combining with Table 7, it can be found that settling times τ of $\varphi \left(T_{sw}\right)$ and $\varphi \left(T_{rw}\right)$ under case I are 140 s and 148 s, respectively, when the base fluid (0%) is used. When the NVF is 10%, the settling time τ of $\varphi \left(T_{sw}\right)$ and $\varphi \left(T_{rw}\right)$ shrink to 109 s and 114 s with 31 s and 34 s drops, respectively. The settling time τ of $\varphi \left(T_{sw}\right)$ and $\varphi \left(T_{rw}\right)$ under case II are 176 s and 186 s, respectively, when the base fluid (0%) is adopted. They decrease to 130 s and 137 s with 46 s and 49 s drops, respectively, when the NVF is 10%. The results indicate that the settling time τ declines with increasing NVF. The changing ratios ζ of $\varphi \left(T_{sw}\right)$ and $\varphi \left(T_{rw}\right)$ in case I drop to 16.67% and 15.32%, respectively, when the base fluid is used. These ratios decrease to 14.93% and 13.39% with declines of 1.74% and 1.93%, respectively, when the nanofluid with a NVF of 10% is adopted. In contrast, these ratios in case II climb to 14.25% and 16.60%, respectively, when the base fluid is used. They decrease to 12.78% and 14.71% with 1.47% and 1.89% drops, respectively, when the NVF is 10%. Thus, it can be summarized that the changing ratios ζ of $\varphi \left(T_{sw}\right)$ and $\varphi \left(T_{rw}\right)$ decrease with the increasing NVF (1–10%). Combining with the analysis above, it can be concluded that using nanofluid to cool the BLSG will decrease the settling time and temperature changing ratios, which improves the transient thermal performance of NBCS. Additionally, such superiority can be enlarged with the increasing NVF (1–10%).

### 4.4. Effect of Nanofluid on BLSG System Power Losses and Efficiency

**Figure 8:**
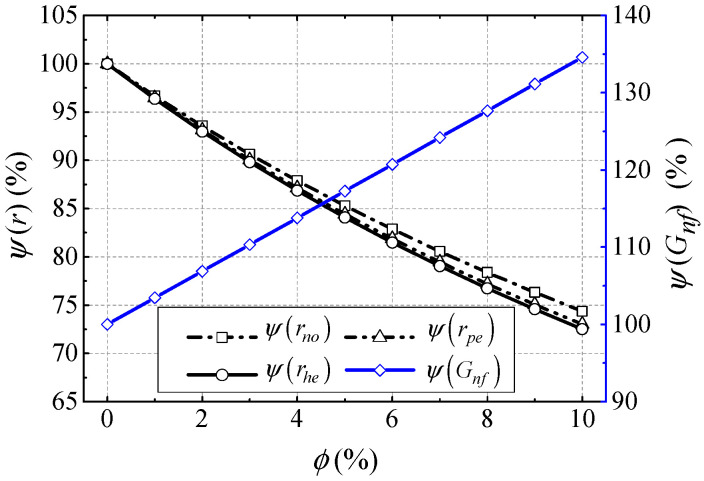
Typical nanofluid flow resistances and mass flow under different NVF.

**Figure 9:**
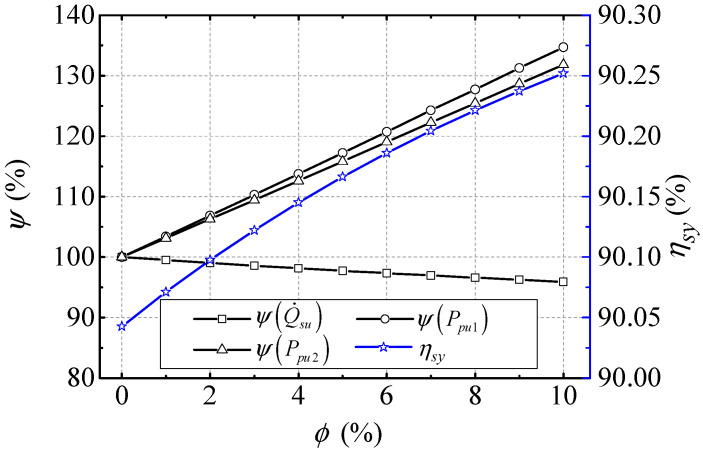
Power loss and efficiency of the NBCS under different NVF.

In order to obtain an accurate analysis of the power losses and efficiency of NBCS, all kinds of power in the BLSG system should be considered. This means that not only the power losses in the BLSG, but also the power consumption in the cooling loop of the NBCS is considered in the power losses and efficiency analysis. The power consumption in the cooling loop mainly occupied the input power of pumps which used to overcome the flow resistances in the cooling system, such as the flow resistances of the BLSG nozzles, pipes, and heat exchanger hot end. Because the nanofluid thermophysical properties influence the flow resistances directly, which eventually affects the input power of pumps, some typical flow resistances in the cooling system under different NVFs are analyzed and normalized in Figure 8. The total power loss in BLSG, input power of pumps, and efficiency of the NBCS are given in Figure 9.

The normalized flow resistances of BLSG nozzles, pipes, and heat exchanger hot end $\psi \left(r_{no}\right)$, $\psi \left(r_{pe}\right)$, and $\psi \left(r_{he}\right)$, respectively, are found linearly decline with the NVF increasing in Figure 8.

Their drops are 25.36%, 27%, and 27.5%, respectively, when the nanoparticle volume fraction is 10%. While the normalized nanofluid mass flow $\psi \left(G_{nf}\right)$ increases with increasing NVF. It rises by ~34.6% at the 10% NVF condition. According to Equations (24) and (25), it can be deduced that it is the increasing nanofluid density contributes to the decrease of flow resistances. On the condition of constant nanofluid VFR, the increasing nanofluid density also leads to the increase of nanofluid mass flow.

In Figure 9, the normalized total power loss in BLSG $\psi \left({\dot{Q}}_{su}\right)$, and the input power of pumps I and II $\psi \left(P_{in,pu1}\right)$ and $\psi \left(P_{in,pu2}\right)$ are illustrated. It can be obtained that $\psi \left(P_{in,pu1}\right)$ and $\psi \left(P_{in,pu2}\right)$ linearly increase with increasing NVF. Their increments are 34.7% and 31.8%, respectively, when NVF is 10%. It means that the input power of pumps have about more than 30% increase under the condition of 10% NVF. While the $\psi \left({\dot{Q}}_{su}\right)$ has a ~4.1% decline under the condition of 10% NVF. The efficiency of the NBCS $\eta_{sy}$ increases from 90.04% to 90.25% with ~0.21% promotion. It can be deduced from Equations (10)–(16) that the increases of pumps I and II input power, $P_{in,pu1}$ and $P_{in,pu2}$, are due to the increase of nanofluid mass flow though the flow resistances decrease. The decrease of BLSG total power loss ${\dot{Q}}_{su}$ is reason for the copper losses decreases which can be found in the Figure 5c. Because of the drop in BLSG total power loss, ${\dot{Q}}_{su}$, is larger than the summation of incensements of $P_{in,pu1}$ and $P_{in,pu2}$, the efficiency of NBCS $\eta_{sy}$ is promoted by ~0.21%. The results show that applying the nanofluid as cooling medium in the NBCS can cause the input power of pumps to increase and the BLSG total power loss to decrease, which eventually result in the total power loss of NBCS decrease and efficiency promotion.

## 5. Conclusions

This paper proposes a nanofluid-based cooling method for a brushless synchronous generator. Detailed thermal performance and efficiency analysis of the nanofluid-based cooling system (NBCS) are delivered. The main results are concluded as follows.

(1) The heat transfer coefficient between the sprayed nanofluid and windings and that between the nanofluid and pipes are increased with the increase of nanoparticle volume fraction (NVF), their increments are 63% and 58%, respectively, when the NVF is 10%. The increasing heat transfer coefficients contribute to the heat dissipation of NBCS.
(2) The steady-state thermal performance of NBCS is improved as the NVF increases. Specifically, when the NVF changes from 0% (base fluid) to 10%, the steady-state temperatures of MG stator and rotor winding are decreased by 33.2 °C and 36.9 °C, respectively, and the MG stator and rotor copper losses are decreased by 7.4% and 8.3%, respectively. The efficiency of BLSG is promoted by ~0.35%.
(3) Since the settling time together with the dynamic changing ratios of the temperature of MG stator and rotor are decreased with the increase of NVF, the transient thermal performance of the NBCS is improved as the increase NVF.
(4) As the NVF increased from 0% to 10%, the input power of the cycling pumps in the NBCS increased more than 30%, while the total power loss in BLSG has a ~4.1% decrease. However, since the power loss reduction in the BLSG is larger than the total increment of the input power of pumps, the efficiency of NBCD still has a slight promotion.

All of our results show that the proposed nanofluid-based cooling method is far superior to the base fluid cooling method. It reveals that the nanofluid-based cooling method has a broad application prospect in the field of thermal control of electrical rotating machine with high power density. It can be expected that the nanofluid-based cooling system also has good thermal properties when the other nanoparticles such as CuO and carbon nanotubes are adopted. Besides, the models and results in this paper are expected to benefit the thermal design process of the thermal management system of the advanced aircraft generator.

## Figures and Tables

**Table 1:** Physical parameters and operating conditions of the NBCS.

Physical Parameter	Value	Initial Operating Condition	Value
voltage $U_{N}$	270 V	Rotate speed n	15000 r/min
Rated current $I_{N}$	240 A	Output power $P_{o}$	65 kW
Pole pairs	6	Coolant volume flow $q_{nf}^{0}$	0.6143 L/s
BLSG Mass $m_{G}$	8.81 kg	Fuel inlet temperature $T_{f}$	65 °C
Winding temperature coefficient $k_{R}$	3.9 × 10^−3^	Fuel mass flow $G_{f}$	0.8437 kg/s

**Table 2:** Thermophysical parameters of Al_2_O_3_ particles and the lubricating oil.

	Density (kg/m^3^)	Specific Heat (J/(kg*K))	Thermal Conductively (W/(m*K))	Viscosity (kg/(m*s))
Al_2_O_3_ particles	3970	750	30	-
Lubricating oil	893	1909	0.14	0.028

**Table 3:** Case arrangement for steady-state analysis.

NVF	Operating Condition
0%, 1%,2%, 3%, 4%, 5%, 6%, 7%,8%, 9%,10%	See in Table 1

**Table 4:** Case arrangement for transient analysis.

Case	Parameters	Initial Value	Final Value	Description	Particle Volume Fraction
Case I	BLSG output power $P_{o}$	65 (kW)	52 (kW)	20% step reduction	0%, 1%, 4%, 7%,10%
Case II	Nanofluid volume flow rate $q_{nf}$	0.6143 (L/s)	0.4914 (L/s)	20% step reduction	0%, 1%, 4%, 7%,10%

**Table 5:** Steady-state performance of the NBCS under base fluid cooling.

Parameters	Value	Parameters	Value
Stator copper loss ${\dot{Q}}_{gsc}^{0}$	1585.7 W	Pipe flow resistance $r_{pe}^{0}$	1.163 × 10^6^ Pa·s/m
Rotor copper loss ${\dot{Q}}_{grc}^{0}$	1816.8 W	Pump I input power $P_{in,pu1}^{0}$	238.9 W
BLSG efficiency $\eta_{G}$	90.46%	Pump II input power $P_{in,pu2}^{0}$	93.8 W
BLSG system efficiency $\eta_{sy}$	90.04%	Heat convection coefficient in pipe $\alpha_{pe}^{0}$	288.1 W/m^2^·K
MG stator winding temperature $T_{sw}$	217.3 °C	Heat convection coefficient of spray $\alpha_{w}^{0}$	817.6 W/m^2^·K
MG rotor winding temperature $T_{rw}$	213.4 °C	Mass flow $G_{nf}^{0}$	0.549 (kg/s)
reservoir oil temperature $T_{roi}$	69.96 °C		

**Table 6:** Heat dissipation of BLSG.

NVF(%)	0%	1%	4%	7%	10%
${\dot{Q}}_{nf}$(W)	5396.35	5383.2	5347.48	5316.39	5289.22
${\dot{Q}}_{le}$(W)	880.96	860.1	803.08	752.66	707.96
${\dot{Q}}_{to}$(W)	6277.31	6143.3	6150.56	6069.05	5997.18

**Table 7:** The settling time and change ratio of the MG stator and rotor winding temperature.

	Cases	Temperature	0%	1%	4%	7%	10%
τ(s)	Case I	$T_{sw}$	140	136	126	117	109
		$T_{rw}$	148	143	132	123	114
	Case II	$T_{sw}$	176	164	154	141	130
		$T_{rw}$	186	179	163	152	137
ξ(%)	Case I	$T_{sw}$	−16.67	−16.46	−15.90	−15.39	−14.93
		$T_{rw}$	−15.32	−15.08	−14.43	−13.83	−13.29
	Case II	$T_{sw}$	14.25	14.09	13.57	13.17	12.78
		$T_{rw}$	16.60	16.41	15.75	15.27	14.71

**Figure A1:**
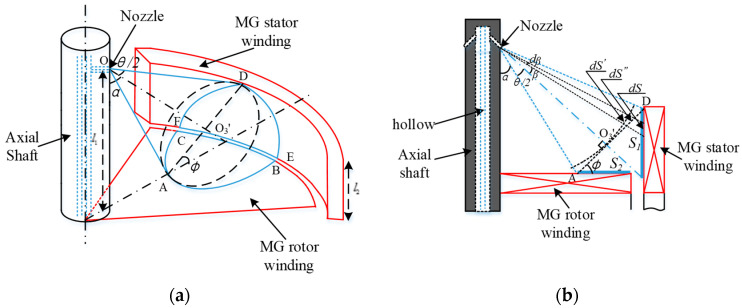
Diagram of the MG stator and rotor winding cooling: (**a**) physical sketch and (**b**) schematic diagram.

## Nomenclature

$A_{du}$	sectional Area of duct (m^2^)	fo	fuel out heat exchanger cold end
$A_{sp}$	Nanofluid spray impact area (m^2^)	gsc	MG stator copper loss
C	specific heat (J/K)	gsi	MG stator iron loss
d	inner diameter (m)	grc	MG rotor copper loss
$d_{32}$	Sauter mean diameter	G	BLSG
f	frequency (HZ)	he	heat exchanger hot end
F	bearing load (N)	HE	Heat exchanger
G	mass flow rate (kg/s)	il	inlet
h	nanofluid entropy (J/kg)	l	Local fraction
I	armature current (A)	le	Leakage heat
$k_{f}$	friction flow resistance coefficient	ma	machine loss
$k_{l}$	Local flow resistance coefficient	nf	nanofluid
$k_{R}$	electrical resistance temperature coefficient	no	nozzle
l	length (m)	nos	Total nozzles
m	mass (kg)	nir	nanofluid in reservoir
Nu	Nusselt number	nis	nanofluid in oil sump
p	pressure (Pa/m^2^)	noh	nanofluid flow out of heat exchanger heat end
$P_{o}$	BLSG output power (W)	oi	Lubrication oil
$P_{in,pu1}$	input power of pump I (W)	ois	oil in sump
$P_{in,pu2}$	input power of pump II (W)	ol	outlet
Pe	Peclet number	os	oil sump
Pr	Prandtl number,	ox	oil in axle shaft
q	volumetric flow rate (L/s)	p	Nano-particles
$\dot{Q}$	power loss (W)	pe1	pipe I
r	flow resistance (Pa*s/m)	pe2	pipe II
R	thermal resistance (K/W)	psc	PE stator copper loss
$R^{\prime }$	electrical resistance (Ω)	psi	PE stator iron loss
Re	Reynolds number	pu1	pump I
T	temperature (°C).	pu2	pump II
$\dot{T}$	temperature gradient (°C/s)	r	MG rotor
V	volumetric flux (L/(m^2^*s))	ri	reservoir inlet
Greek symbols		ro	reservoir outlet
α	convective heat transfer coefficient (W/(m^2^*°C/))	rw	MG rotor winding
λ	thermal conductively (W/(m*k))	s	Mg stator
ω	angular velocity (rad/s)	sp	spray
η	efficiency	sh	BLSG shell
ρ	Density (kg/m^3^)	st	BLSG installation structure
μ	viscosity (kg/(m*s))	su	sum
χ	phase number	sw	MG stator winding
ϕ	Nano-particle volume fraction	sy	NBCS system
σ	thermal diffusivity (m^2^/s)	to	total
subscript		w	winding
a	ambient	x	axle shaft
ai	air in BLSDCG		
ce	Heat exchanger cold end	I−II	Between I and II, where I and II include nf, sw, rw, s, r, x, sh, a, st, nih, ai, HE and fic
erc	ME rotor copper loss		
eri	ME rotor iron loss		
esc	ME stator copper loss	III(IV)	The IV of III, III include ta, pu1, p1, he and ce, IV include *il* and *ol*.
fi	fuel in heat exchanger cold end		

## Appendix A

*(a) Power Losses Calculation*

${\dot{Q}}_{su}$ is the summation of copper losses, iron losses, and mechanical loss in BLSG. The calculation method of the three kinds of power loss is given as follows.

(1) Copper loss: The copper loss is caused by the currents flowing in the stator and rotor wingdings in operation. The winding copper loss is calculated as Equation (A1) [46,47]:

(A1) $${\dot{Q}}_{cu}=nI_{w}^{2}{R^{\prime }}_{ef}$$

where n is the phase number, $I_{w}$ is the RMS value of single phase current flowing the winding, and ${R^{\prime }}_{ef}$ is the effective electrical resistance. The electrical resistance is dramatically effect by its temperature and can be described as

(A2) $${R^{\prime }}_{ef}=\left[1+k_{R}(T_{w}-20)\right]{R^{\prime }}_{20}$$

where $k_{R}$ is the temperature coefficient, $T_{w}$ is the temperature of winding, and ${R^{\prime }}_{dc}$ is the electrical resistance in 20 ℃.

(2) Iron loss: the iron loss composes of three basic losses, which are hysteresis loss, eddy current loss, and the excess loss [48]; the heat resulted from the iron loss can be obtained by Equation (A3).

(A3) $${\dot{Q}}_{ir}=m\left[k_{h}fB_{m}{}^{\alpha }+k_{e}{\left(fB_{m}\right)}^{\alpha }+k_{a}{\left(fB_{m}\right)}^{1.5}\right]$$

where m is the mass of the iron material, a is a constant coefficient which depends on the material,f is the frequency, $B_{m}$ is the maximum value of the flux density, α is an exponent which equals to 1.7–2.0 for silicon steel lamination, and $k_{h}$, $k_{e}$, and $k_{a}$ are the coefficients which depend on the material.

(3) Machine loss: The machine loss is a consequence of windage loss and bearing friction loss, it can be described by Equation (A4). The windage loss in BLSG is produced from the friction between the rotating surface and the surrounding gas. Equation (A5) can be used to calculate the windage loss [46,49,50]: According to SKF (1994) [29], the bearing friction losses can be obtained by Equation (A6).

(A4) $${\dot{Q}}_{m}={\dot{Q}}_{wl}+{\dot{Q}}_{be}$$

(A5) $${\dot{Q}}_{wl}=\frac{k_{f}\rho_{a}\pi \omega^{3}d_{r}^{4}l_{r}}{8}$$

(A6) $${\dot{Q}}_{be}=0.5\omega k_{be}Fd_{be}$$

where ${\dot{Q}}_{m}$ is the machine loss, ${\dot{Q}}_{wl}$ is the windage loss, ${\dot{Q}}_{be}$ is the bearing fraction loss, $k_{f}$ the is the friction coefficient, $\rho_{a}$ is the density of the air in the BLSG, ω is the rotating speed, $d_{r}$ is the diameter of the rotor, $l_{r}$ is the rotor core length, $k_{be}$ is the friction coefficient (typically 0.001–0.005), F is the bearing load, and $d_{be}$ is the inner diameter of the bearing.

*(b) Temperature Model of Key Parts in BLSG*

In order to calculate the MG stator and rotor winding temperature in Equation (2), the temperature of other key parts in BLSG, such as the MG stator and rotor, should be obtained. In this paper, the other parts are the MG stator, MG rotor, BLSG shell, axle shaft, and air in BLSG. The temperature models of those parts are given as

(A7) $$\left\{ \begin{array}{c} c_{s}m_{s}{\dot{T}}_{s}=\frac{\lambda_{s-sw}}{\delta_{s-sw}}A_{s-sw}\left(T_{sw}-T_{s}\right)+\frac{\lambda_{s-sh}}{\delta_{s-sh}}A_{s-sh}\left(T_{sh}-T_{s}\right)+\alpha_{ai}A_{s-ai}\left(T_{ai}-T_{s}\right)+{\dot{Q}}_{gsi}\\ c_{r}m_{r}{\dot{T}}_{r}=\frac{\lambda_{r-rw}}{\delta_{r-rw}}A_{r-rw}\left(T_{rw}-T_{r}\right)+\frac{\lambda_{r-x}}{\delta_{r-x}}A_{r-x}\left(T_{x}-T_{r}\right)+\alpha_{ai}A_{r-ai}\left(T_{ai}-T_{r}\right)\\ c_{sh}m_{sh}{\dot{T}}_{sh}=\frac{\lambda_{s-sh}}{\delta_{s-sh}}A_{s-sh}\left(T_{s}-T_{sh}\right)+\frac{\lambda_{sh-x}}{\delta_{sh-x}}A_{sh-x}\left(T_{x}-T_{sh}\right)+\alpha_{a}A_{sh}\left(T_{a}-T_{sh}\right)+\frac{\lambda_{st}}{\delta_{st}}A_{st}\left(T_{st}-T_{sh}\right)+{\dot{Q}}_{psc}+{\dot{Q}}_{esc}+{\dot{Q}}_{psi}\\ c_{x}m_{x}{\dot{T}}_{x}=\frac{\lambda_{r-x}}{\delta_{r-x}}A_{r-x}\left(T_{r}-T_{x}\right)+\frac{\lambda_{sh-x}}{\delta_{sh-x}}A_{sh-x}\left(T_{sh}-T_{x}\right)+{\dot{Q}}_{eri}+{\dot{Q}}_{erc}+{\dot{Q}}_{ma}\\ c_{ai}m_{ai}{\dot{T}}_{ai}=\alpha_{ai}A_{s-ai}\left(T_{s}-T_{ai}\right)+\alpha_{ai}A_{r-ai}\left(T_{r}-T_{ai}\right) \end{array}\right.$$

where the subscripts s, r, sh, x, and ai represent the MG stator, MG rotor, BLSG shell, axle shaft, and air in BLSG, respectively, and λ, δ, and A are the equivalent conductivity coefficient, equivalent thickness, and contact area, respectively, the symbol ‘−’ in the subscript means between its front and back objects, for example $A_{sh-x}$ is the contact area between BLSG shell and axle shaft. $\alpha_{ai}$ is the convective heat transfer coefficient between air in BLSG and BLSG parts and $\alpha_{a}$ is the convective heat transfer coefficient between ambient and NBSC.

*(c) Pipe Temperature Model*

The temperature dynamic of pipe can be derived by Equation (A8).

(A8) $$c_{pe1}m_{pe1}{\dot{T}}_{pe1}=\alpha_{pe}A_{pe}\left(T_{nip1}-T_{pe1}\right)+\alpha_{a}A_{pe}\left(T_{a}-T_{pe1}\right)$$

where $c_{pe1}$ and $m_{pe1}$ are the specific heat capacity and mass of pipe I, respectively, and ${\dot{T}}_{pe1}$ is the temperature gradient of pipe I.

*(d) Reservoir Temperature Model*

The temperature dynamic of reservoir is given by

(A9) $$c_{re}m_{re}{\dot{T}}_{re}=\alpha_{re}A_{re}\left(T_{nir}-T_{re}\right)+\alpha_{a}A_{re}\left(T_{a}-T_{re}\right)$$

where $c_{re}$ and $m_{re}$ are the specific heat capacity and mass of reservoir, respectively, and ${\dot{T}}_{re}$ is the temperature gradient of reservoir.

*(e) Analysis of the Convective Heat Transfer Coefficients between the Nanofluid and Windings*

In order to analysis the spray cooling process clearly, the physical sketch of the MG stator and rotor winding cooling is given in Figure A1a, based on which the schematic diagram is obtained as Figure A1b. As shown in Figure A1b, the spray cone angle is θ. Line $O{O^{\prime }}_{3}$ is the center line of spray cone angle, and it is perpendicular to line AD. The cooling area of MG stator and rotor winding are represented as *S*_1_ and *S*_2_, respectively.

It can be easy obtain that the convective heat transfer coefficient is depended on the volumetric flux under the same condition. M. Visaria et al. [51] had analyzed the relationship between the volumetric flux and spray inclination. According to their study, the volumetric flux for spherical surface $S^{\prime }$ in Figure A1b is calculated as:

(A10) $$V^{\prime }=\frac{q}{2\pi L^{2}\left[1-\cos\left(\theta /2\right)\right]}$$

where $V^{\prime }$ is the volumetric flux for spherical surface, q is the coolant flow rate, and L is the distance from orifice to surface $S^{\prime \prime }$.

The volumetric flux across the surface $S_{1}$ and $S_{2}$ are given as

(A11) $$V_{1}=V^{\prime }\frac{dS^{\prime }}{dS_{1}}$$

(A12) $$V_{2}=V^{\prime }\frac{dS^{\prime }}{dS_{2}}$$

where $V_{1}$ and $V_{1}$ are the volumetric fluxes across surfaces $S_{1}$ and $S_{2}$, respectively.

*S*_1_ and *S*_2_ are considered to be axially symmetric about line $O{O^{\prime }}_{3}$ when the effect of radian on the surface $S_{1}$ is ignored. Thus, the equation can be obtained as follows

(A13) $$\frac{dS^{\prime }}{dS_{1}}\approx \frac{dS^{\prime }}{dS_{2}}$$

According to Equations (A10)–(A13), it can be obtained that

(A14) $$V_{1}\approx V_{2}$$

It can be obtained that the convective heat transfer coefficient on MG stator winding $\alpha_{w1}$ equals to that of MG rotor winding $\alpha_{w2}$ when $V_{1}$ equals to $V_{2}$ on the same spray condition.

(A15) $$\alpha_{w1}\approx \alpha_{w2}$$

Thus, in the paper, $\alpha_{w}$ is applied to represent both MG stator and MG rotor winding convective heat transfer coefficient.
